# Strategic positioning of immunization at the heart of Africa’s health and development agenda

**DOI:** 10.1080/21645515.2025.2599628

**Published:** 2025-12-11

**Authors:** Charles S. Wiysonge, Duduzile Ndwandwe, Chinwe Iwu-Jaja, Chukwudi A. Nnaji, Shingai Machingaidze, Abdu A. Adamu, Andre Arsene Bita Fouda, Gregory D. Hussey

**Affiliations:** a Cochrane South Africa, South African Medical Research Council, Cape Town, South Africa; b Centre for Evidence-Based Health Care, Department of Global Health, Stellenbosch University, Cape Town, South Africa; c Vaccine-Preventable Diseases Programme, World Health Organization Regional Office for Africa, Brazzaville, Congo; d Technical Department, Malaria Consortium, London, UK; e Africa Strategy and Engagement, Coalition for Epidemic Preparedness Innovations, London, UK; f Vaccines for Africa Initiative, School of Public Health, University of Cape Town, Cape Town, South Africa; g Institute of Infectious Disease and Molecular Medicine, University of Cape Town, Cape Town, South Africa

**Keywords:** Africa, immunization, primary health care, implementation science, sustainable financing

## Abstract

Immunization is one of the greatest public health achievements of all times, preventing millions of deaths and making major improvements in child survival in Africa. Beyond protecting against infectious diseases, vaccines foster educational attainment, productivity, and poverty reduction, while mitigating antimicrobial resistance and economic shocks. However, each year more than 9.5 million children in the World Health Organization African Region remain under-vaccinated, resulting in recurrent outbreaks of vaccine-preventable diseases. In 2024 alone, 59 outbreaks were reported across the region, including measles, variant polioviruses, meningitis, cholera, and yellow fever. To meet growing demands amid constrained resources, we propose three strategic shifts to reimagine immunization as a catalyst for resilient health systems and equitable development in Africa: (1) transitioning from vertical delivery models to integrated approaches embedded in primary health care; (2) expanding success metrics beyond coverage to include equity, resilience, and process indicators, supported by implementation science and digital innovations; and (3) reframing immunization as a whole-of-society development priority anchored in political leadership, sustainable financing, and regional vaccine manufacturing. By embracing these shifts, Africa can transform immunization into a driver of health system strengthening, equity, and sustainable development.

Immunization is one of the greatest public health achievements of all times.^1,2^ Since the inception of the Expanded Programme on Immunization (EPI) in 1974, the evidence of its impact in Africa has been unequivocal. It has prevented over three billion disability-adjusted life years and accounted for over half the improvement in child survival on the continent.^1^ Immunization not only saves lives but also enhances human capital formation. It contributes to healthy childhood development, reinforces educational attainment, enhances productivity, and reduces the risk of antimicrobial resistance (AMR).^3–6^ Every dollar invested in childhood immunization yields returns of more than 50 times.^2^

Immunization programs have served as platforms for delivering broader health services.^3^ Routine immunization sessions in mobile and outreach sites, as well as supplementary immunization activities, have extended maternal and child health, nutrition, malaria prevention, and other services to underserved communities.^5–7^ Facility-based routine immunization services have also served as key touchpoints for the co-delivery of family planning, malaria prevention, and other interventions. These co-benefits amplify the developmental dividend of every vaccine dose delivered.

However, there is no room for complacency. As we commemorate substantial progress with immunization in Africa, the path forward is as complex as it is urgent. Although regional coverage for most vaccine doses in 2024 had been restored to at least pre-pandemic 2019 levels, this incremental improvement falls short of recommended targets.^8,9^ In addition, half of the world’s under-vaccinated children live in Africa.^8,9^ At the same time, immunization programs across Africa are under mounting pressure to deliver more with fewer resources.

The expansion of immunization schedules, growing expectations for data integration, and the imperative for increasing community engagement, all place significant burdens on under-resourced immunization programs. Rwanda’s experience introducing new vaccines illustrates both the promise and complexity of this task.^10^ Between 2009 and 2011, the country successfully introduced pneumococcal conjugate, rotavirus, and human papillomavirus (HPV) vaccines by grounding decisions in local disease surveillance, feasibility assessments, and cost-effectiveness analyses. Policymakers relied on routine health data to prioritize high-impact vaccines and address delivery bottlenecks, while ensuring equitable reach across rural and urban populations. Yet, they faced challenges such as limited supply chain capacity, increased staff workload, and the need for community engagement; which were addressed through phased introductions, strong political commitment, and contextualized engagement mechanisms.^10^ Similarly, recent evidence from outside Africa shows how genotype-specific data informed the selection of the most suitable rotavirus vaccine for national introduction in Indonesia, underscoring the value of evidence-informed decision-making in new vaccine adoptions.^11^ These examples highlight the importance of aligning national immunization policies with local epidemiology, system capacity, and societal context.

The challenges faced by immunization programs reflect deep systemic issues such as weak health systems, understaffing, supply chain vulnerabilities, conflict and population displacement, pervasive vaccine hesitancy, and limited domestic financing.^12–15^ Addressing these barriers requires bold and systemic changes. The African immunization community must embrace a broader and bolder vision; one that transcends technical delivery and anchors immunization within national development priorities. Thus, we propose three strategic shifts to reimagine immunization as a driver of health system transformation and development impact in Africa. Although our analysis draws primarily from the World Health Organisation African Region, the proposed strategies are broadly relevant to all countries in Africa and beyond; with contextual adaptation to local governance and health system realities.^14^

The first strategic shift entails a transition from siloed immunization programs – including polio eradication – to integrated delivery models embedded in primary health care and multisectoral platforms. This means, for example, aligning vaccine delivery with maternal and child health, nutrition, surveillance (including for AMR), WASH (water, sanitation, and hygiene), and school health services. It also calls for harmonized investments in health workforce, supply chains, and information systems.

Countries should adopt diversified and contextualized delivery strategies to reach every person and community. These should combine facility-based services with mobile and outreach sessions, house-to-house visits, and engagement of the private (formal and informal) health sector. Community-based actors and local leaders should be empowered to drive demand and trust. Institutionalizing life-course immunization – extending protection from children to adolescents, adults, and healthcare workers – is critical to building and sustaining population immunity. For sustainable integration, immunization must be embedded in cross-cutting health system strengthening efforts, including community healthcare worker programs and health sector reforms. A truly integrated approach positions immunization as a cornerstone of resilient, equitable, and responsive health systems.

However, operational integration may bring challenges. Healthcare workers may face heavier workloads, competing priorities, and additional reporting requirements. Addressing these challenges will require practical and contextualized solutions.^16^ These include task-shifting and task-sharing with community healthcare workers; synchronized microplanning across immunization, maternal and child health, nutrition, and other services; and the use of readiness assessments to sequence and pace new vaccine introductions. Digital tools such as electronic stock management systems, tablet-based reporting platforms, and simplified dashboards can help reduce administrative workload and streamline data entry for healthcare workers. Strengthening supply chain optimization through redesigned distribution routes, buffer stock planning, and real-time temperature monitoring also helps prevent bottlenecks. Together, these measures reduce frontline burden and support the effective integration of immunization into primary health care.

The second strategic shift requires a move from focusing only on coverage metrics to include equity, resilience, and process indicators. Measuring success only by national coverage masks inequalities. Programs must adopt context-relevant indicators of equity, resilience, and implementation quality. This will enhance rapid data-driven learning and adaptation and pave the way for robust use of implementation science tools for subnational tailoring.^17^

Immunization equity is a crucial indicator of the strength and fairness of national health systems. Beyond national averages which may obscure subnational inequities in access, a true measure of success must be the ability to make immunization services available to children in fragile settings, informal settlements, nomadic communities, and other settings where immunization services are currently “hard-to-reach.”^12^ Equity, resilience, and implementation process indicators must be routinely monitored to inform real-time decision-making and adaptation.^18^ This requires harmonized, digital, and interoperable data systems that provide individual-level tracking, enable triangulation of data sources, and support targeted outreach to under-vaccinated children.

Equity indicators may include the proportion of under-vaccinated children reached in informal urban settlements or conflict zones; resilience indicators could measure recovery time from vaccine stock-outs or service interruptions; and process indicators might track the frequency and timeliness of micro-plan reviews and data-use meetings. Routine monitoring of these metrics through harmonized data systems will enable real-time learning and adaptive management.

Reaching under-vaccinated children in conflict-affected or insecure areas requires contextualized delivery models.^19–21^ Proven approaches include deploying mobile health teams, integrating immunization into humanitarian outreach clinics, and using short-duration vaccination sessions when security windows open. Partnership with local non-governmental organizations, faith-based organizations, and community volunteers who remain resident in conflict-affected and insecure zones enables continuity when external access is limited. Geospatial mapping, satellite imagery, and community informant networks would help identify displaced or mobile populations, while cross-border vaccination points would support those in transit. In contexts of prolonged conflict, the use of humanitarian buffer stock and negotiated humanitarian access corridors are critical for maintaining delivery of immunization and other services.^19–21^

Embedding implementation research into immunization programs will accelerate adaptive learning, enable early identification of bottlenecks, and generate local solutions. Continuous monitoring and iterative feedback loops are critical for sustaining progress.^17,18^ Peer learning and regional knowledge exchanges can facilitate the scale-up of successful innovations across settings with similar challenges, reinforcing a culture of continuous improvement and mutual accountability.^22^ Digital transformation must be systematized to support efficient supply chains, real-time monitoring of key metrics, and predictive analytics. These tools are essential for planning and improving service delivery, particularly in decentralized and dynamic contexts.

In practice, real-time monitoring should focus on a defined set of metrics such as vaccine stock levels, frequency of immunization session cancellations, cold chain temperature excursions, zero-dose identification, timeliness of outreach sessions, and micro-plan updates. Many countries use platforms such as District Health Information Software 2 (DHIS2), electronic Logistics Management Information Systems (eLMIS), and Short Message Service (SMS)- or Unstructured Supplementary Service Data (USSD)-based reporting tools to consolidate these data.^23^ In low-connectivity settings, offline-enabled tablets and store-and-forward systems allow data to be captured during sessions and uploaded once connectivity is available. Weekly data review meetings at facility and district levels would facilitate rapid interpretation and corrective action. These approaches would ensure that even in remote, conflict, or fragile settings, programs have access to near-real-time information that guides local decision-making.

The third strategic shift is to transform immunization from a health sector intervention to a whole-of-society development priority. It should be elevated to a development priority in each country and reframed as a strategic investment in human capital. It is essential for achieving multiple Sustainable Development Goals including health, education, and poverty reduction. Ministries of Finance, Planning, Education, and Communication, as well as local governments, civil society, the private sector, and other societal actors should all champion immunization as a foundation for economic growth and social stability.

The Education sector is particularly important, as school-based platforms offer effective channels for delivering vaccines to adolescents, strengthening health literacy, and building long-term trust in public health.^16^ The Communications and Information Technology sector would also play a vital enabling role through the development of interoperable digital systems, secure digital identities, social listening tools, and platforms that counter misinformation on social media and the internet. These sectors collectively would help to reinforce the societal foundations required for high and sustained vaccination coverage. Context-specific investment cases should be developed to communicate the true social and economic value of immunization, and the projected gains and contributions to the national economy.

Countries such as Rwanda^24,25^ and South Africa^26,27^ have begun integrating immunization within primary health care reforms or life-course frameworks, demonstrating the feasibility of the strategic shifts. However, these and other countries face challenges such as limited fiscal space, workforce shortages, and fragmented data systems.^7^ These lessons highlight both the promise and the complexity of implementing the proposed strategic shifts continent-wide.

Achieving these transformative shifts requires collective action. Political leadership is critical because governance matters.^28^ African Heads of State, First Ladies, and First Gentlemen must champion immunization as a catalyst for development and equity. The Addis Declaration on Immunization, endorsed in 2017 by Heads of State, remains a powerful framework for aligning political will with national action on immunization.^29^

Sustainable immunization financing must be a shared responsibility.^30^ Global donors, development banks, and the private sector have important roles to play. However, countries must lead the way through domestic resource mobilization, efficient program management, and long-term investment strategies. Countries must institutionalize immunization financing within national budgets and legal frameworks to secure long-term sustainability. Innovative financing mechanisms – including insurance schemes, performance-based funding, and blended finance models – should be explored to reduce donor dependency.^31^ Domestic and external resource mobilization must be matched by efficient program management and transparent accountability mechanisms.

Innovation and data must be harnessed for impact; using real-time data systems, geospatial tools, and behavioral insights to transform how services are planned, delivered, and evaluated. Partnerships across organizations, sectors, and borders must evolve to meet complexity, with stronger coordination mechanisms and shared accountability. Investments in regional vaccine manufacturing, regulatory harmonization, and supply chain digitization are essential for ensuring timely access to safe, affordable, and locally produced vaccines. Meeting at least 60% of Africa’s vaccine needs through regional production by 2040 should be a shared priority.^32^

Community trust and engagement must be at the center of all efforts. Culturally sensitive and evidence-based communication strategies that reflect local realities and social norms are critical for building lasting vaccine confidence. Behavioral and social data should inform targeted social and behavior change interventions, co-created with communities themselves.^33^ Embedding implementation research within national immunization programs should become the norm.^34,35^

Africa’s immunization journey has been one of extraordinary progress, but it is far from over.^7^ The next five years are pivotal. With the Immunization Agenda 2030 reaching midterm and new global and continental strategies emerging,^36–38^ this is a defining moment for decisive action. Immunization should be recognized as a vital instrument for building health system resilience, driving human capital development, and advancing equity and inclusion across the continent. We call on governments, communities, partners, and all stakeholders to unite behind a bold new vision – one that places immunization at the heart of Africa’s health and development agenda. In reimagining immunization as a development priority, we are envisioning a healthier, more prosperous and brighter future for Africa.

To realize this bold new vision, Africa must: (1) integrate immunization into people-centered primary health care; (2) expand monitoring and data-informed learning to encompass equity, resilience, and process indicators; and (3) reframe immunization as a whole-of-society investment driving human capital and development. Together, the three shifts can transform immunization into a true engine of resilience, equity, and prosperity across the African continent.
